# Oncometabolites and the response to radiotherapy

**DOI:** 10.1186/s13014-020-01638-9

**Published:** 2020-08-14

**Authors:** Kexu Xiang, Verena Jendrossek, Johann Matschke

**Affiliations:** Institute of Cell Biology (Cancer Research), University Hospital Essen, University of Duisburg-Essen, Virchowstrasse 173, 45147 Essen, Germany

**Keywords:** Oncometabolites, Ionizing radiation, DNA repair, Epigenetic regulation

## Abstract

Radiotherapy (RT) is applied in 45–60% of all cancer patients either alone or in multimodal therapy concepts comprising surgery, RT and chemotherapy. However, despite technical innovations approximately only 50% are cured, highlight a high medical need for innovation in RT practice. RT is a multidisciplinary treatment involving medicine and physics, but has always been successful in integrating emerging novel concepts from cancer and radiation biology for improving therapy outcome. Currently, substantial improvements are expected from integration of precision medicine approaches into RT concepts.

Altered metabolism is an important feature of cancer cells and a driving force for malignant progression. Proper metabolic processes are essential to maintain and drive all energy-demanding cellular processes, e.g. repair of DNA double-strand breaks (DSBs). Consequently, metabolic bottlenecks might allow therapeutic intervention in cancer patients.

Increasing evidence now indicates that oncogenic activation of metabolic enzymes, oncogenic activities of mutated metabolic enzymes, or adverse conditions in the tumor microenvironment can result in abnormal production of metabolites promoting cancer progression, e.g. 2-hyroxyglutarate (2-HG), succinate and fumarate, respectively. Interestingly, these so-called “oncometabolites” not only modulate cell signaling but also impact the response of cancer cells to chemotherapy and RT, presumably by epigenetic modulation of DNA repair.

Here we aimed to introduce the biological basis of oncometabolite production and of their actions on epigenetic regulation of DNA repair. Furthermore, the review will highlight innovative therapeutic opportunities arising from the interaction of oncometabolites with DNA repair regulation for specifically enhancing the therapeutic effects of genotoxic treatments including RT in cancer patients.

## Background

Radiotherapy (RT) is commonly used to treat cancer, especially solid tumors. RT uses the local application of ionizing radiation (IR) to target and to kill cancer cells with high precision and has beneficial effects on loco-regional control, overall survival and cure rates in various tumor types. In fact, the therapeutic potential of RT alone and in multimodal combinations with surgery, chemotherapy, and targeted drug therapy has increased considerably during the past decades [1]. However, advanced cancers are characterized by pronounced radioresistance, leading to local relapse, whereas co-irradiation of normal tissues may lead to toxicity, thereby limiting the maximal applicable RT dose. The risk of adverse effects also limits therapy intensification efforts by combining RT with any other cancer therapy, RT dose escalation, so that local recurrence of primary tumors and distant metastases remain leading causes of death in many cancer patients [2, 3].

The broad use of RT as standard treatment option in the therapy of solid human tumors is based on its ability to damage cellular macromolecules, particularly DNA double strand breaks (DSB) thereby effectively inducing growth arrest, and cell death in irradiated tumor cells [4]. However, high intrinsic, microenvironment-mediated, and adaptive radioresistance of solid human tumors, remain major obstacles to successful RT. For example, the cytotoxic efficacy of radiotherapy relies on the local availability of molecular oxygen (O_2_) in the tumor tissue during treatment delivery for the generation of reactive oxygen species (ROS) and the fixation of RT-induced DNA damage. Consequently, an acute severe decrease in O_2_ levels (“tumor hypoxia”) by insufficient O_2_ supply, increased O_2_ demand, or both, confers direct resistance by decreasing oxidative stress and therapy-induced cell killing [5].

Important molecular determinants of intrinsic and acquired radioresistance are i) the cellular capacity to detoxify radiation-induced ROS and ii) the capacity to perform efficient repair of RT-induced DNA damage, particularly the most lethal DSBs [6]. Although DSBs stand for small proportion of DNA lesions induced by RT [7], they are an enormous challenge. Therefore, cells developed various mechanisms to ensure survival amongst others by distinct pathways for DSB repair, e.g. non-homologous end-joining (NHEJ), homologous recombination repair (HRR), or alternative end-joining (alt-EJ) [8, 9]. Consequently, genetic abnormalities that enhance the capacity of cancer cells to perform DSB repair via NHEJ, HRR, or alt-EJ promote cancer cell survival exposed to genotoxic therapies and enhance radioresistance [10–12]. Instead, genetic abnormalities leading to defects in the DNA damage response (DDR) and DNA DSB repair pathways such as early onset Breast cancer 1/2 (BRCA1/2) enhance sensitivity to DNA-damaging treatments, such as chemotherapy and radiotherapy, and generate specific vulnerabilities to inhibitors of complementary DSB repair pathways in so-called synthetic lethality approaches [13–17].

Interestingly, emerging evidence indicates that factors beyond genetic defects in core proteins of DDR and DSB repair, e.g. microenvironmental cues [5, 18–20] or deregulated expression or mutations in chromatin modifiers [21–24] or metabolic enzymes [10, 25] can also promote DSB repair defects in cancer cells with important therapeutic implications. Moreover, the ability of cancer cells to maintain cellular redox homeostasis and high antioxidant capacity as part of the metabolic reprogramming during malignant progression has relevance to radioresistance [10, 25–29]. Finally, metabolic adaptation of cancer cells to adverse conditions in the tumor microenvironment or treatment-induced stress can promote acquired radioresistance offering additional targets for tumor-specific radiosensitization [10, 25, 29–33]. However, one caveat of using metabolic inhibitors in cancer therapy remains the large molecular heterogeneity within and between different tumors, highlighting the urgency to develop reliable biomarkers for patient stratification.

Taken together, there is a high medical need for novel and effective biology-based strategies for a tumor-specific radiosensitization. Research in molecular radiobiology and radiation oncology therefore aims to define genetic and environmental factors that mediate intrinsic and adaptive radiation resistance in individual tumors, as well as cancer cell specific defects that may allow for a tumor-specific radiosensitization on an individual basis, including heterogeneous tumors.

## Main text

### Role of genetic and epigenetic alterations of DSB repair for radioresistance

NHEJ and HRR are considered as the two major DSB repair pathways [8]. The cell cycle-independent NHEJ is a very fast but error prone DSB repair machinery, whereas HRR is only active if the template DNA for repair is present (G2/S cell cycle phase) [9]. Both pathways rely on a certain set of proteins Therefore, it is not surprising that documented genetic alterations in gene expression or signaling of these DSB repair proteins influences efficiency of DSB repair and thus the sensitivity of cancer cells to RT [22, 23, 34–37]. However, DNA repair is also regulated by epigenetic enzymes, both on the chromatin and the DNA level [24, 38–44]: The molecular details of the interplay between epigenetics and DSB repair has been described by others and will therefore not be described in detail here (for details see reviews by Dabin J et al. [43], Lahtz C et al. [42] and Gong F et al. [43].).

### Therapeutic strategies using synthetic lethality with genetic defects in DSB repair

With the advent of the genomics era evidence is now accumulating that cancer cells are characterized by pronounced genomic instability and also more frequently harbor defects in DNA repair proteins than expected, including core proteins of DSB repair [5, 16, 45]. Interestingly, such cancer-cell specific alterations in a core DSB repair protein enhance vulnerability to drugs interfering with the respective alternative DSB repair pathways, and this effect can be further enhanced when such drugs are combined with DNA damage-inducing treatments, e.g. RT or genotoxic chemotherapy [16, 45–47]. The underlying concept of synthetic lethality had been developed for tumors with loss-of function mutations in HR-genes BRCA1 or BRCA2 [45, 47]. BRCA-mutant tumors harbor defects in HR-repair (“BRCAness” or “HRness”) and this is synthetically lethal with inhibition of Poly(ADP-Ribose)-Polymerase (PARP)-dependent DNA repair pathways using PARP-inhibitors [45–47]. However, cancer cells can also develop resistance against treatments with DNA repair inhibitors highlighting the need for novel therapeutic approaches to prevent or overcome resistance and for the identification and validation of robust biomarkers predicting response or resistance to inhibitors of the DNA damage response and DNA repair [46, 48]. In this context, intrinsic or pharmacologically induced metabolic defects in HRR may offer elegant opportunities for improving the outcome of cancer therapy beyond synthetic lethality approaches with PARPi, e.g. by using inhibitors of NHEJ (e.g. inhibitors of DNA-dependent serine/threonine protein kinase (DNA-PK)). As outlined above, oncogene-induced or drug-induced metabolic constraints of HRR will also enhance radiosensitivity of cancer cells. Here, the suggested higher relative importance of HRR for the repair of DNA damage induced by particle therapy (e.g. carbon ions [49], and proton beam therapy [50–53]) may even offer potential future opportunities for the stratification of patients with oncometabolite-rich tumors towards particle therapy, or combining particle therapy approaches with metabolic drugs inducing HRR defects during therapy.

In this context, a concept of metabolic induction of DNA repair defects could represent an elegant opportunity for improving the outcome of RT allowing for a cancer-cell specific radiosensitization of cancer cells with genetic defects or pharmacologic inhibition of end-joining (EJ) dependent pathways and even synergize with potential differences in the biology of DNA damage induced by irradiation with proton beams compared to irradiation with gamma-ray photons or X-ray photons.

### Genetic defects in cancer cells and accumulation of oncometabolites

Various studies demonstrate that nuclear and mitochondrial DNA-encoded mitochondrial genes are mutated in cancer and that this phenomenon is connected with poor clinical outcome and prognosis [54, 55]. However, not all cancers exhibit mitochondrial dysfunction and it should be emphasized that the complete loss of mitochondrial function is detrimental for cancer cells [56]. Identification of cancer-associated mutations in genes with impact on the cellular metabolism has drawn great attention during recent years [57–62]: Some of these mutations introduce abnormal production of certain metabolites with relevance to cancer progression, termed “oncometabolites”. Researchers have unveiled the abnormal production of 2-hydroxyglutarate (2-HG), succinate and fumarate in cancer and linked their critical roles to cellular metabolic transformation and biological processes [63–66]. Reprogramming of cancer cell metabolism, as a consequence of genetic and epigenetic alterations, can influence the metabolic phenotype of cancer cells and the production of oncometabolites, thereby enhancing downstream oncogenic cascades [67]. Furthermore, by altering anti-immune response and activating dormant and therapy-resistant cancer cells oncometabolites can also modulate tumor progression, cancer aggressiveness and tumor repopulation after radio- or chemotherapy [68–72].

#### Succinate

Succinate is a critical metabolite of the tricarboxylic acid cycle (TCA) and plays an important role in cellular metabolic processes [73]. Succinate dehydrogenase (SDH) is an enzyme complex composed of four subunits (SDHA, SDHB, SDHC, SDHD), and is also termed mitochondrial complex II of the mitochondrial electron transport chain (ETC) [74, 75]. Defects in SDHB, SDHC, SDHD, but not SDHA, have been linked to disturbed function of complex II in the mitochondria [76] and impaired oxidation of succinate to fumarate. Furthermore, a loss-of-function mutation of SDH and the accompanied overproduction of succinate have been linked to the onset of cancer [65, 66] as well as to tumor repopulation after radio- or chemotherapy (as reviewed in [71]). However, the proper SDH function requires participation of oxidized FAD^+^ and NAD^+^ as cofactors, which are short of supply in cancer cells due to mitochondrial dysfunction [77]. Succinate accumulation facilitated angiogenesis by stimulation of succinate receptor 1 (SUCNR1) and accompanied up-regulation of vascular endothelial growth factor (VEGF) expression [78, 79]. Succinate accumulation also activated pathways connected to epithelial-to-mesenchymal transition (EMT), tumor migration, and invasion [80–83], and this might contribute to treatment-induced tumor repopulation and cancer relapse [68, 71].

As expected, SDH deficiency was linked to metabolic reprogramming in cancer cells, and promoted a glycolytic, pseudo-hypoxic phenotype. Genetic knockdown of SDHB in hepatocellular carcinoma (HCC) cell lines, resulted in decreased expression of Complex III and IV of the ETC and increased acidity of the cytoplasm suggesting a switch of cancer cells from mitochondrial respiration to glycolysis as main energy source, known as Warburg effect [84, 85]. The occurrence of a pseudo-hypoxic phenotype induced by succinate accumulation highlights an additional role of this oncometabolite in the modulation of immune responses e.g. promoting the release of the pro-inflammatory chemokine IL-1-β by innate immune cells, such as bone-marrow-derived macrophages (BMDMs) [86]. Interestingly, it has been demonstrated that RT-induced changes in miRNA-expression (miRNA-210, miRNA-31, miRNA-378) modulate the expression of SDH leading to succinate accumulation [71, 87–91]. Even more important, it has been proposed that miRNA-regulated succinate accumulation might promote tumor repopulation and cancer relapse [71].

#### Fumarate

Similar to succinate, fumarate is an important metabolite of the TCA cycle. Loss of function of fumarate hydratase (FH) was linked to overproduction of fumarate, and gave rise to hereditary leiomyomatosis and renal cell cancer (HLRCC) [65, 66]. High fumarate levels can adjust the balance of biochemical reactions in which this oncometabolite plays a role either as substrate or product. For instance, it was demonstrated, that fumarate accumulation impacts the conversion of succinate to fumarate in the TCA cycle, leading to disturbance of SDH-related mitochondrial respiration [92]. Fumarate-accumulation also gave rise to metabolic reprogramming towards argininosuccinate-accumulation via reversal of the urea cycle [93]. Notably, FH-mutated cells require a constant supply of exogenous arginine to keep the urea cycle active and are disabled when arginine is short-of-supply, thereby creating a cancer-specific vulnerability. Similarly, accumulation of fumarate promotes overproduction of adenylosuccinate by reversal of adenylosuccinate lyase (ADSL) within the purine nucleotide cycle (PNC) [93]. In contrast to succinate, fumarate also alters the post-translational modification of cysteine residues of several proteins, called succination. Succination induced by FH-deficiency led to inhibition of mitochondrial respiration, activated antioxidant response, and tumor growth [92, 94–96]. Furthermore, FH-deficient cells reprogram their metabolism in the direction of aerobic glycolysis to provide energy [97]. Thereby, part of carbons from glucose are diverted toward the pentose phosphate pathway (PPP) to maintain redox homeostasis [96].

Furthermore, FH, like SDH, is also regulated by RT-induced miRNA-378 resulting in fumarate accumulation, and might thereby contribute to processes triggering tumor repopulation [71, 98].

#### 2-hydroxyglutarate (2-HG)

Accumulation of the 2-HG enantiomers L2-HG or D2-HG can occur under the following conditions: i) as pathologic metabolites in hypoxic cancer cells produced by lactate dehydrogenase (LDH) or malate dehydrogenase (MDH), respectively, [99–101]; or ii) as “oncometabolites” as a consequence of gain-of-function mutations in the genes coding for isocitrate dehydrogenase 1 or 2 (IDH1 or IDH2) [102, 103]. Herein, LDH and MDH seem to be the primary enzymes responsible for L2-HG generation in hypoxia [99–101], overproduction of D2-HG has been linked to gain-of-function mutations in IDH [101, 102]. Importantly, 2HG was proposed as a potential biomarker for monitoring therapy-response in IDH-mutant glioblastoma patients [104]. Moreover, accumulation of L2-HG has also been observed in renal cell carcinoma as well as in children with defects in ETC components, respectively [105–107]. Accumulating evidence further indicates that L2-HG mediates inhibition of the cellular differentiation process of mouse hematopoietic stem cells [68, 108]. Additionally, 2-HG reprograms nuclear cancer stemness program by impairing histone demethylation thereby lowering the energy barriers separating non-stem and stem-cells [70, 109]. Furthermore, tumor-cell derived D2-HG is taken up by T cells and reprograms nuclear factor of activated T cells (NFAT) transcriptional activity and polyamine biosynthesis, resulting in suppression of T cell activity [72]. These findings highlight a sophisticated crosstalk between cancer cells and tumor-infiltrating immune cells where the oncometabolites modify or inhibit the function of tumor infiltrating immune cells and thereby favor cancer cell proliferation, tumor expansion, and metastasis formation [72, 110] with potential relevance to radiotherapy [69].

### Therapeutic perspectives

**Fig. 1:**
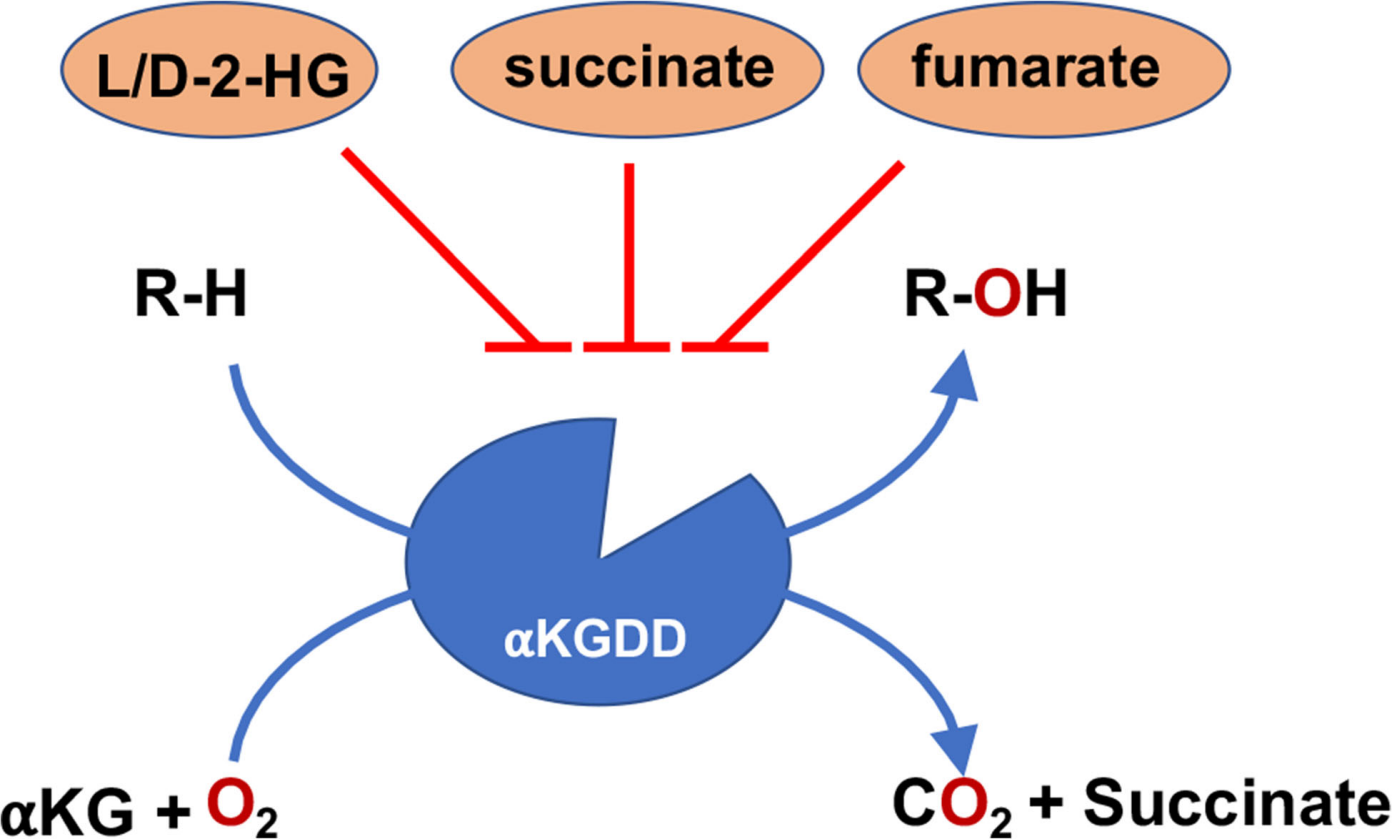
Schematic representation of how oncometabolites inhibit αKG-dependent dioxygenases (αKGDDs). 2-HG, succinate and fumarate are antagonists to αKG and broadly inhibit αKG-dependent dioxygenases (αKGDDs). αKGDDs use O_2_ and αKG as co-factors to perform a range of oxidation reactions gaining succinate, CO_2_ and hydroxylated target molecule. αKG = α-Ketoglutarate, L/D-2-HG = L/D-2-hydroxyglutarate, R = target molecule

Interestingly, the oncometabolites 2-HG, succinate and fumarate described above act as competitive inhibitors of α-Ketoglutarate (KG)-dependent dioxygenases (αKGDD) [111]. The family of αKGDDs use O_2_ and αKG as co-factors to perform a range of oxidation reactions e.g. modification of chromatin or regulation of protein stability, e.g. of hypoxia-inducible factors [112] (Fig. 1). Interestingly, various epigenetic enzymes belong to families of α-KG-dependent enzymes, e.g. α-KG-dependent histone lysine demethylase (KDM) and ten eleven translocation (TET) DNA demethylases with documented regulatory functions in DSB repair [16, 24, 39, 40, 113, 114] of the above oncometabolites will impair DNA repair by inhibition of KDMs and TET DNA demethylases and the resulting histone/DNA hypermethylation [24, 115–118]. The oncometabolite-dependent regulation of αKG-dependent epigenetic enzymes with impact on DNA repair offers new and exciting avenues for a cancer cell-specific radiosensitization and improved radiotherapy outcome, as outlined in the following paragraphs.

#### Succinate

**Fig. 2:**
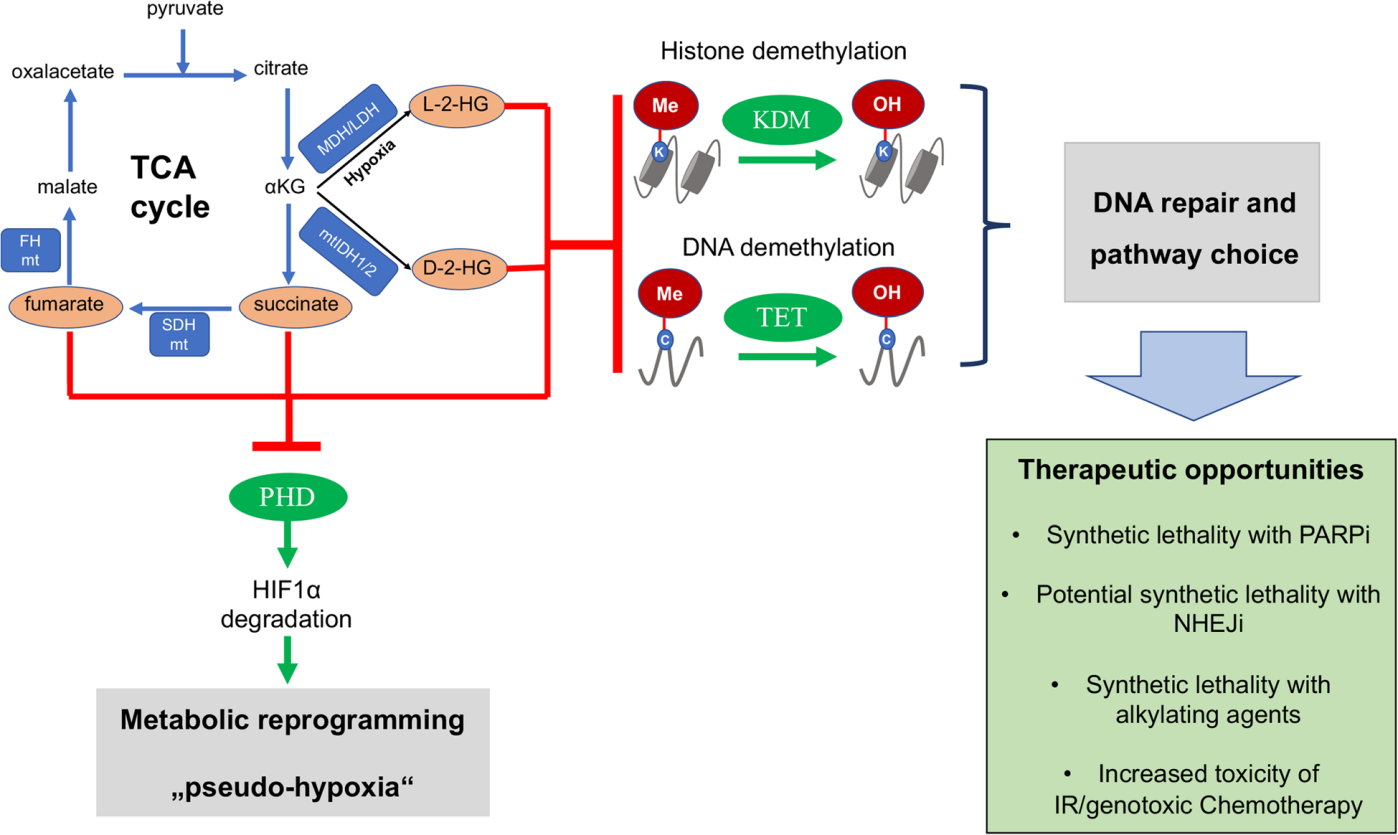
Schematic representation of how oncometabolites can modulate radiosensitivity. The indicated oncometabolites (orange) can accumulate as a consequence of mutations of TCA cycle enzymes or environmental cues, such as hypoxia and even without oncogenic mutations e.g. by pharmacologic inhibition of SLC25A1. 2-HG, succinate and fumarate induce metabolic reprogramming and a “pseudo-hypoxic phenotype” via stabilisation of HIF1α. Moreover, indicated oncometabolites are competitive inhibitors of the αKG-dependent KDM and TET families of epigenetic enzymes, thereby modulating DNA repair and pathway choice and offer novel therapeutic opportunities with IR. αKG = α-Ketoglutarate, C = cytosine, K = Lysin, L/D-2-HG = L/D-2-hydroxyglutarate, mt = mutant, Orange colour represents increased metabolite levels

The above-mentioned succinate effects on regulation of EMT, metastasis, metabolic reprogramming and tumor repopulation are known as critical determinants of radiosensitivity [71, 119, 120]. SDH-deficient cells displayed competitive inhibition of several α-KGDDs, due to progressive succinate accumulation [80]. In more detail, succinate accumulation competitively inhibits α-KG-dependent dioxygenase, a ten eleven translocation (TET) enzyme, which is responsible for the oxidation of 5-methylcytosines (5mCs) and thereby promotes DNA demethylation in normal cells [121]. Therefore, accumulation of succinate was associated with inhibition of TETs resulting in a decrease of 5mC oxidation and DNA hypermethylation [121, 122]. In line with these observations, SDH-mutated gastrointestinal tumor samples revealed a histone hypermethylation, presumably as a consequence of KDM inhibition [123]. We speculate that inhibition of KDMs by succinate accumulation may result in HRR suppression as described for 2-HG-accumulation, thereby rendering cancer cells vulnerable to PARP inhibitors [24, 115, 116] or alkylating agents (Fig. 2).

#### Fumarate

Similarly, fumarate accumulation in FH mutated cells, induced epigenetic regulation of DNA/histone demethylases, with related downstream effect of cancer cells [94, 122, 124]. Interestingly, recent studies suggest a translocation of FH into the nucleus upon DNA damage: nuclear FH led to local production of fumarate that caused inhibition of histone H3K36 demethylation; since histone H3K36 demethylation is an important step in DDR, as it promotes binding of NHEJ proteins and thereby facilitates DNA repair, nuclear FH impaired DSB repair [125]. Furthermore, FH loss-of-function mutation conferred resistance to DNA damage upon IR and promoted early mitotic entry after IR by suppressing checkpoint maintenance [126]. We assume that fumarate accumulation in FH-deficient cells might also inhibit HRR by acting on KDMs, leading to increased endogenous DNA damage and over-sensitivity to PARP-inhibitors [116] or alkylating agents (Fig. 2).

#### 2-HG

Generally, high cellular 2-HG levels have been associated with malignant progression and sensitivity to RT [16, 127], presumably through inhibition of αKGDDs. Cancer cells with mutant IDH and accompanied 2-HG accumulation are characterized by increased sensitivity to DNA-damaging treatments, e.g. IR or alkylating agents [102, 114]. Importantly, elegant recent work revealed that 2-HG accumulation induced a defect in HRR reminiscent of BRCA1/2-deficient tumors as it made the respective cells vulnerable to PARP-inhibitors; interestingly the authors were able to link this effect to inhibition of αKG-dependent dioxygenases of the lysine demethylase family, KDM4A and KDM4B, and thus to epigenetic regulation of DSB repair [16, 113, 128, 129]. In line with this observations, recent studies further associated the function of KDMs to regulation of HRR and radioresistance of lung cancer patients [24, 115, 130]. Moreover, a recently published study by Sulkowski and coworkers has dissected some mechanistic aspects of the link between 2-HG and DSB repair on the level of KDM4B mediated H3K9me3 histone 3 lysine 9 trimethylation (H3K9me3) near DNA breaks [129]. In more detail, 2-HG mediated KDM4B inhibition resulted in an increase of H3K9me3 at loci surrounding DNA breaks. The alteration of the histone marks interfered with proper recruitment of TIP60 and ATM, thereby reducing end resection and impairing downstream DNA repair [129]. This study emphasizes the pivotal role of 2-HG-mediated suppression of HRR and provides an excellent explanation for the relationship between oncometabolites, the DNA damage response and DSB repair [129]. However, 2-HG accumulation also inhibited alkylation repair homolog (ALKBH) DNA repair enzymes leading to enhanced sensitivity to alkylating agents [114, 115].

Taken together, the inhibitory effects of oncometabolites in the process of DSB repair represents a tumor-specific vulnerability offering opportunities for a tumor-specific radiosensitization (Fig. 2).

### Tumor hypoxia and context-dependent vulnerabilities

Exposure to hypoxia leads to a pronounced metabolic adaptation, e.g. inhibition of the TCA cycle and oxidative phosphorylation (OXPHOS) as well as up-regulation of glycolysis, respectively [100, 131]. Activation of glycolysis under conditions of O_2_-deprivation is mostly regulated by activation of the hypoxia-inducible factor 1 (HIF-1) which induces broad metabolic reprogramming to balance O_2_ demand and provision [131, 132]. Elegant work has revealed the various roles of HIF1 in radioresistance [5, 19, 61]. Of note, overproduction of the oncometabolites 2-HG, succinate or fumarate induced a pseudo-hypoxic phenotype by HIF1α-stabilization under normoxic conditions [121, 122] through inhibition of αKGDD prolyl hydroxylases (PHD) responsible for hydroxylation and subsequent degradation of HIF1α [24, 115, 130]. Thus, oncometabolite-induced HIF1α-mediated metabolic reprogramming towards a pseudo-hypoxic phenotype might be associated with increased resistance to certain cancer therapies, but offer potential vulnerabilities associated with the resulting DNA repair defects. As an example, inhibition of mitochondrial citrate carrier SLC25A1 induced the accumulation of 2-HG in cancer cells and this was associated with impaired repair of radiation-induced DNA damage [25]. Metabolic reprogramming of cancer cells exposed to acute or chronic cycling severe hypoxia with intermittent reoxygenation supported RT resistance by increasing cellular antioxidant capacity and up-regulation of the mitochondrial citrate carrier protein SLC25A1 and the dicarboxylate carrier protein SLC25A10 [10, 25, 29]. It is tempting to speculate that pharmacologic induction of oncometabolite accumulation might by a suitable strategy to induce epigenetic tuning of DNA repair pathways during therapy to enhance the efficacy of RT.

## Conclusions and outlook

Exciting recent observations highlight a role of oncometabolites in the regulation of antioxidant capacity, mitochondrial respiration, and DSB repair with impact on cancer cell sensitivity to genotoxic chemotherapy and RT. For instance, TCA-cycle derived oncometabolite-accumulation competitively suppressed the function of αKGDDs with relevance to epigenetic regulation of DNA repair, e.g. KDMs and TETs, resulting in DNA/Histone hypermethylation. However, the biochemical binding properties of the different oncometabolites suggest different flavors of specificity for αKGDDs with relevance to DNA repair [121, 133]. While fumarate-accumulation promotes protein succination and thereby triggers a plethora of biological changes that may synergise with or counteract αKGDD inhibition, 2-HG-accumulation suppresses the function of KDMs affecting HRR [16, 61, 129]. Furthermore, fumarate and 2-HG evoke opposite effects on mTOR signaling, with consequences for the development of certain tumor types [134, 135] and potential indirect influence on radiation sensitivity. Additional studies suggest that specific environmental or nutritional circumstances may even promote oncometabolite-accumulation in the absence of the underpinning oncogenic mutations. This might be relevant for the reported link between oncometabolite and cancer stemness or suppression of the anti-tumor immune response thereby shaping the tumor immune microenvironment as suggested by others. Furthermore, RT-induced changes in miRNA-expression might mediate therapy-induced oncometabolite accumulation and tumor repopulation [71]. Finally, other oncometabolites such as Sarcosine, a N-methyl derivative of the amino acid glycine observed in prostate cancer, also increase DNA methylation, yet their impact on DNA repair and radiosensitivity remains to be determined. Taken together, a better mechanistic understanding of the accumulation of oncometabolites, the metabolic communication between mitochondria and the nucleus, and of the metabolic regulation of epigenetic enzymes with impact on DNA repair will allow to use genetic defects and the altered interplay between cell metabolism, epigenetic enzymes and DNA repair for tumor-specific synthetic lethality in combination with DNA repair inhibitors tumor-specific radiosensitization, or both in the future. The identification of biochemical nodes for oncometabolite induction may even allow us to use metabolic inhibitors, such as mitochondrial transporters, for temporal induction of oncometabolites during radiotherapy in tumors without genetic oncometabolite-induction. We assume that combining systematic cell biology and radiobiology investigations with mathematical modeling of the obtained results will allow to identify critical epigenetic regulators of radiosensitivity and the discovery of metabolic targets for tumor-specific radiosensitization.

## Methods

Studies were identified via searching electronic databases e.g. Pubmed, Web of Science with key words: radiotherapy, ionizing radiation, radiation therapy, DNA damage response, DDR, DNA repair, epigenetic regulation, epigenetic modulation, double-strand break, DSB, oncometabolite, 2-hydroxyglutarate, 2-HG, fumarate and succinate for publications in English. Studies and reviews related with ionizing radiation and/or metabolism were included. Publications focusing on novel cancer therapies, such as hormonal therapy, were taken out of consideration. To be more reliable, conclusions from different publications had been cross examined. Unpublished materials were not included in this review.

## Data Availability

Not applicable.

## Abbreviations

αKGDD: α-ketoglutarate-dependent dioxygenase
2-HG: 2-hydroxyglutarate
5mCs: 5-methylcytosines
ADSL: adenylosuccinate lyase
ADSL: adenylosuccinate lyase
ALKBH: Alkylation Repair Homolog
alt-EJ: alternative end-joining
BRCA1/2: early onset Breast cancer ½
DDR: DNA damage response
DNA-PK: DNA-dependent serine/threonine protein kinase
DSB: DNA double strand break
EJ: end-joining
EMT: epithelial-to-mesenchymal transition
ETC: Electron transport chain
FH: fumarate hydratase
HCC: hepatocellular carcinoma
HIF: hypoxia-inducible factor
HLRCC: hereditary leiomyomatosis and renal cell cancer
HRR: homologous recombination repair
IDH: isocitrate dehydrogenase
IR: Ionizing radiation
KDM: histone lysine demethylase
LDH: lactate dehydrogenase
MDH: malate dehydrogenase
NHEJ: non-homologous end-joining
O2: molecular Oxygen
OXPHOS: oxidative phosphorylation
PARP: Poly(ADP-Ribose)-Polymerase
PHD: prolyl hydroxylase
PPP: pentose phosphate pathway
ROS: reactive oxygen species
RT: Radiotherapy
SDH: Succinate dehydrogenase
SUCNR1: succinate receptor 1
TCA: tricarboxylic acid cycle
TET: ten eleven translocation DNA demethylase
VEGF: vascular endothelial growth factor
αKG: α-Ketoglutarate
